# Tackling cisplatin resistance in ovarian cancer: what can we do?

**DOI:** 10.20517/cdr.2021.59

**Published:** 2021-07-08

**Authors:** Paola Perego

**Affiliations:** Molecular Pharmacology Unit, Department of Applied Research and Technological Development, Fondazione IRCCS Istituto Nazionale dei Tumori, Milan 20133, Italy.

Ovarian cancer is a complex disease mainly consisting of epithelial cancer which comprises various histological subtypes. The most frequent one is high-grade serous ovarian carcinoma. The prognosis of this disease is poor because most women are diagnosed with advanced disease. Although many patients respond to first-line Pt-based therapy, the development of resistance limits the efficacy of therapy. Novel therapeutic strategies to improve the efficacy of ovarian carcinoma treatment are emerging from both basic and translational research efforts. In this Special Issue, approaches to improve the armamentarium to fight ovarian carcinoma are presented, thereby providing an overview of selected aspects of ovarian cancer biology that can be exploited to improve treatment. In this context, a re-evaluation and optimization of already tested strategies such as intra-peritoneal therapy together with a deep investigation of the recently acquired knowledge on factors (e.g., E3 ubiquitin ligases) and features [Cancer Stem Cells (CSCs)] contributing to drug resistance may provide useful insights to tackle cisplatin resistance.

Takahashi *et al*.^[1]^ reviewed the role of CSCs in ovarian cancer recurrence, describing recently acquired results in the field with particular emphasis on therapeutic options. They highlighted that ovarian CSCs, besides being associated with poor prognosis, may underlie recurrence. In fact, repetitive cycles of chemotherapy have been shown to enrich the tumor for CSCs. Multiple pathways have been reported to be activated in CSCs (e.g., Wnt and hedgehog signaling), thereby opening various opportunities for CSC-targeting therapies including drug combinations with chemotherapeutic agents. In this regard, the identification of CSCs through appropriate markers is key.

Meng *et al*.^[2]^ reviewed the available knowledge on the emerging role of E3 ubiquitin ligases in drug resistance of ovarian carcinoma with a focus on the available inhibitors and their potential for clinical use. As a large family of enzymes with more than 600 members, several E3 ubiquitin ligases participate in key cellular processes for survival of cancer cells, thereby contributing to chemoresistance. Regulation of drug resistance can occur through a variety of mechanisms, implying the control of the levels of proteins involved in apoptosis or DNA repair. For example, CRL4Cul4/DDR1, a Cullin RING E3 ubiquitin ligase, regulates drug resistance of ovarian cancer cells by targeting the anti-apoptotic protein BIRC3. UBR5, a HECT domain ubiquitin E3 ligase found to be a poor prognostic marker in serous ovarian carcinoma, modulates the accumulation of damage by suppressing RNF168, another E3 ubiquitin ligase containing a RING finger.

Rinne *et al*.^[3]^ reviewed recent advances in targeting the PI3K/AKT/mTOR pathway in ovarian cancer, particularly in relation to drug resistance. The available evidence supports that the pathway is deregulated in ovarian cancer due to alterations mainly involving mutation or amplification of PI3KCA, amplification of AKT, and deletion or inactivation of PTEN. Several inhibitors of PI3K, mTOR, and AKT have been tested in early phase clinical trials, but none of them has progressed to late phase trials for ovarian cancer patients. Despite this, agents inhibiting key components of this pathway hold promise particularly in combination with chemotherapeutic or other targeted agents. In this regard, the targeting of eukaryotic initiator factor 4E (eIF4E), as highlighted by Romagnoli *et al*.^[4]^, appears to open novel opportunities in the field. Because eIF4E binds to the 5’7 methylguanosine mRNA cap thereby selecting the mRNA to be translated, it is crucial in controlling the translation of proteins, including those promoting cancer aggressiveness. Indeed, eIF4E has been shown to be over-expressed in ovarian cancer and other cancer types. Thus, compounds that mimic the mRNA cap structure may be attractive drug candidates for ovarian cancer.

In the search for novel treatment options against ovarian cancer, intra-peritoneal drug administration still appears to be a promising route. Muggia and Bonetti^[5]^ highlighted how this approach may be useful in ovarian cancer management given the need to minimize the emergence of platinum resistance. Tumors bearing homologous recombination defects highly sensitive to platinum compounds are also prone to early peritoneal dissemination. Thus, patient selection for clinical studies should take into account *BRCA* mutations or other alterations resulting in homologous recombination deficiency. The historical overview reported by the authors provides suggestions to address new question through novel trials aimed at overcoming platinum drug resistance using the intra-peritoneal route.

In summary, this Special Issue includes review articles focusing on selected fields of investigation which offer the opportunity to consider the advancements in preclinical research and biology of ovarian cancer in a translational perspective.

## DECLARATIONS

### Copyright

© The Author(s) 2021.
